# Association between intake of red and processed meat and the risk of heart failure: a meta-analysis

**DOI:** 10.1186/s12889-019-6653-0

**Published:** 2019-03-29

**Authors:** Kun Cui, Yabin Liu, Lingjun Zhu, Xia Mei, Ping Jin, Yuhui Luo

**Affiliations:** 1 Department of Cardiology, Chongqing General Hospital, No.1, Youyi Road, Yuzhong district, Chongqing, 400016 People’s Republic of China; 2 Department of Cardiology, Second Affiliated Hospital of Zhejiang University, Hangzhou, People’s Republic of China

**Keywords:** Dietary, Processed meat, Red meat, Heart failure, Meta-analysis

## Abstract

**Background:**

Many studies have assessed the association between consumption of red and processed meat and the risk of heart failure, but the results are not consistent. This meta-analysis aimed to comprehensively evaluate the relationship between intake of red and processed meat and the risk of heart failure.

**Methods:**

Databases of Web of Knowledge, PubMed, and Wan Fang Med Online were retrieved up to date of August 31st, 2017. Suitable publications were identified through using the defined inclusion criteria. The summarized relative risk (RR) with the corresponding 95% confidence interval (CI) was calculated.

**Results:**

Six scientific literatures were included in this study. In comparison with the lowest category, the summarized RR and 95% CI of the highest category of processed meat intake for heart failure risk was 1.23 (95% CI = 1.07–1.41, *I*^2^ = 58.9%, *P* = 0.045). A significant connection between processed meat intake and heart failure was identified among the Europeans (RR = 1.33, 95% CI = 1.15–1.54), but not the Americans. Yet few of essential association was found between heart failure risk and red meat intake (RR = 1.04, 95% CI = 0.96–1.12).

**Conclusions:**

Findings of this meta-analysis indicated that the highest category of processed meat intake, other than red meat intake, correlated with an increased risk of heart failure.

**Electronic supplementary material:**

The online version of this article (10.1186/s12889-019-6653-0) contains supplementary material, which is available to authorized users.

## Background

Due to tremendous advance in modern medicine, the morbidity and mortality of heart failure reduces markedly. However, it remains a great burden to patients and their families [1]. It has been reported that 23 million people are suffering from the heart failure around the world [1, 2], which led to approximately 5% hospital admissions of all adults [3]. Epidemiologic studies indicated that genetic background is one of established risk factors for heart failure patients [4, 5]. Furthermore, other potential risk factors, such as dietary habits including fish consumption [6–8], vitamin D supplementation [9], antioxidant vitamin supplementation [10], fasting plasma glucose [11], and chocolate consumption [12, 13] are all investigated to show associations with heart failure risk. Consumption of meat, including processed and red meat, has also been studied in relationship to risk of heart failure. Generally, processed meat contains high amounts of sodium, which may increase the risk of heart risk through its effect on blood pressure [14]. In addition, recent researches have shown that the intake of dietary meat no matter processed meat or red meat may be a risk factor for heart failure, but some inconsistency has been aroused between the consequences of the studies. The objective of this manuscript was to explore the association between intake of processed meat and red meat and risk of heart failure.

## Methods

The current study was performed in accordance with the guidelines of Meta-analysis of Observational Studies and the Statement of Preferred Reporting Items for Systemic Meta-analysis [15].

### Literature search

Studies were identified from the databases of Web of Knowledge, PubMed, and Wan Fang Med Online up to date of August 31st, 2017. The following research terms were used: ‘heart failure’ and ‘red meat’ (unprocessed) and/or ‘processed meat’. Two investigators conducted this systematic research independently.

### Inclusion and exclusion criteria

The inclusion criteria for studies in the present report were as following: (1) observational studies; (2) reporting the association between the intake of red meat and/or processed meat and the risk of heart failure; (3) the relative risk (RR) with the corresponding 95% confidence interval (CI) were available; (4) human study; (5) articles published in English or Chinese languages.

### Data extraction and quality assessment

The following relevant information were extracted: the first author’s name, year of publication, region for conducting the study, study type, age, cases and participants, duration of follow-up, exposure classification, and confounders adjusted for and RR with 95% CI for the association between the intake of processed meat and red meat and the heart failure risk. The Newcastle-Ottawa-Scale (NOS) was used for evaluating the quality of each study [16].

### Statistical analysis

RR with 95% CI was applied to combine the overall results [17]. Heterogeneity among the included studies was calculated with I^2^ statistical method [18]. The random effects model was employed [19]. Meta-regression analysis was performed to interpret the high between-study heterogeneity [19]. Furthermore, sensitivity analysis was utilized to assess the stability of the results when a single study was removed. Potential publication bias was examined by using the Egger regression asymmetry test [20]. A two-sided *P* < 0.05 indicated statistically significant difference. Statistical analysis was performed by utilizing the stata 12.0 software (STATA, College Station, TX, USA).

## Results

### Search results

**Fig. 1:**
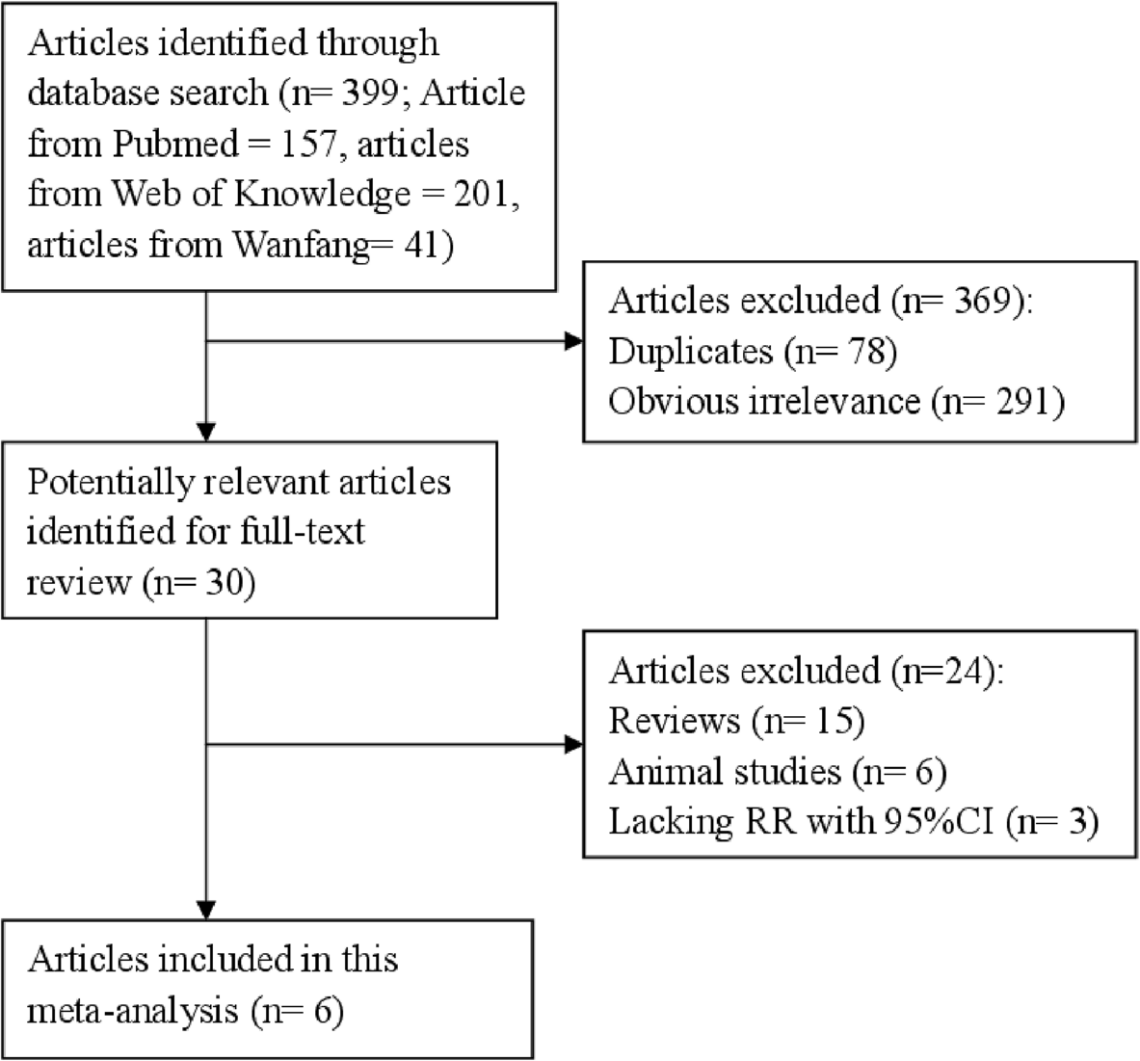
Study selection process for this meta-analysis

Figure 1 displays the flow diagram of this study. The initial screening identified 399 articles from databases. After excluding duplications from the different databases, 321 articles were reviewed based on their titles and abstracts. Twenty-nine articles were further full text reviewed. The final analysis in this report included a total of 6 articles [21–26]. All of the studies had a prospective cohort design. Five studies were conducted to assess the association between the intake of processed meat and the risk of heart failure, while 5 studies evaluated the relationship between the red meat intake and the risk of heart failure. Three studies were performed in North America and the other three studies were carried out in Europe. All of the suitable studies included 9630 heart failure cases and 134,863 participants. All of the six studies had relatively high quality (over 6 stars), with an average NOS score of 7.67. The characteristics of the included studies are summarized in Table 1.

**Table 1:** Characteristics of studies on dietary processed meat or red meat and heart failure risk

Study, year	Country (study)	Quality scores	Participants (cases)	Age (years)	Follow-up duration	Amounts of meat intake	RR (95% CI)	Method of heart failure ascertainment	Method of dietary assessment	Adjustment for covariates
Ashaye A 2011	United States (Physicians’ Health Study)	8	21,120 (1204)	54.6	19.9	Red meat Quartile 1 Quartile 2 Quartile 3 Quartile 4 Quartile 5	Red meat 1 1.02(0.85–1.22) 1.08(0.90–1.30) 1.17(0.97–1.41) 1.24(1.03–1.48)	Obtained through yearly questionnaires; the HF diagnoses had been previously confirmed with the use of the Framingham criteria[	Semiquantitative food frequency questionnaire (19 items)	Adjusted for age, aspirin assignment, smoking, alcohol consumption, cereal consumption, parental history of MI prior to age 60 y, exercise and body mass index, and prevalent diabetes, coronary heart disease, atrial fibrillation, and hypertension at 12 months post randomization.
Del Gobbo 2015	United States (Cardiovascular Health Study)	7	4490 (1380)	72	21.5	Processed Quartile 1 Quartile 2 Quartile 3 Quartile 4 Quartile 5 Red Quartile 1 Quartile 2 Quartile 3 Quartile 4 Quartile 5	Processed 1 1.01(0.95–1.27) 1.11(1.84–1.47) 1.12(0.85–1.48) 1.21(0.92–1.60) Red 1 0.77(0.65–0.91) 0.92(0.78–1.09) 0.91(0.77–1.07) 0.94(0.80–1.10)	1) diagnosis by a treating physician; 2) HF symptoms (shortness of breath, fatigue, orthopnea, or paroxysmal nocturnal dyspnea) plus signs (edema, rales, tachycardia, gallop rhythm, or displaced apical impulse) or supportive findings on echocardiography, contrast ventriculography, or chest radiography; and 3) medical therapy for HF, defined as diuretics plus either digitalis or a vasodilator.	Using a validated 99-item food frequency questionnaire	Adjusted for age, sex, race, enrollment site, education, annual income, total kcal expended, walking pace, smoking, and alcohol intake.
Kaluza J 2014	Sweden (Swedish Men)	8	37,035 (2891)	45–79	11.9	Processed < 25.0 g/d 25.0–49.9 50.0–74.9 ≥75.0 Red < 25.0 g/d 25.0–49.9 50.0–74.9 ≥75.0	Processed 1 1.09(1.00–1.19) 1.09(0.97–1.23) 1.28(1.10–1.48) Red 1 0.96(0.86–1.07) 0.99(0.88–1.11) 0.99(0.87–1.13)	Events of HF were defined according to the International Classification of Diseases and Related Health Problems, 10th Revision (ICD code I50 and I11.0).	Diet was assessed with a 96-item food-frequency questionnaire	Adjusted for age, education, smoking status, and pack-years of smoking, body mass index, total physical activity, aspirin use, supplement use, family history of myocardial infarction at < 60 y, and intake of energy and consumption of alcohol, whole grain products, fruit, vegetable, and fish.
Kaluza J 2015	Sweden (Swedish Mammography Cohort)	7	34,057 (2806)	48–83	13.2	Processed < 25 g/d 25–49.9 ≥50 Red < 25 g/d 25–49.9 ≥50	Processed 1 1.09(0.99–1.19) 1.30(1.05–1.60) Red 1 0.88(0.81–0.96) 1.00(0.89–1.13)	Events of HF were defined according to the International Classification of Diseases and Related Health Problems, 10th Revision (ICD code I50 and I11.0).	Diet was assessed with a 96-item food-frequency questionnaire	Adjusted for age, education, smoking status, and pack-years of smoking, body mass index, total physical activity, aspirin use, supplement use, family history of myocardial infarction at < 60 y, and intake of energy and consumption of alcohol, whole grain products, fruit, vegetable, and fish.
Nettleton JA 2008	United States (Atherosclerosis Risk in Communities study)	8	14,153 (1140)	45–64	13	Processed or Red	Highest vs. lowest categories 1.07(0.97–1.17)	Incident HF cases were identified through review of county death certificates and local hospital discharge lists and defined according to the International Classification of Diseases Codes (ICD-9 or ICD-10).	Using a 66-item semiquantitative food frequency questionnaire	Adjusted for energy intake, plus demographics: age, sex, race/center, education level lifestyle factors: physical activity level, smoking, and drinking status, and prevalent disease status: cardiovascular disease, diabetes, and hypertension.
Wirth J 2016	Germany (European Prospective Investigation into Cancer and Nutrition-Potsdam)	8	24,008 (209)	35–65	8.2	Processed Quartile 1 Quartile 2 Quartile 3 Quartile 4 Quartile 5	Processed 1 1.73(1.05–2.80) 1.40(0.86–2.31) 1.57(0.92–2.65) 2.04(1.17–3.55)	(a) self-report, (b) death certificates (diagnosis I50 of ICD-10 as underlying cause of death), (c) link to the hospital information system of the major hospital in the Potsdam area and (d) validation of participants who suffered from incident myocardial infarction or reported the use of medications typical for the treatment of HF.	Using a semi-quantitative, self-administered food frequency questionnaire	Adjusted for sex and energy intake, stratified for age, educational degree, physical activity and smoking status.

Abbreviations: *RR* relative risk; *CI* confidence interval

### Processed meat intake and risk of heart failure

**Fig. 2:**
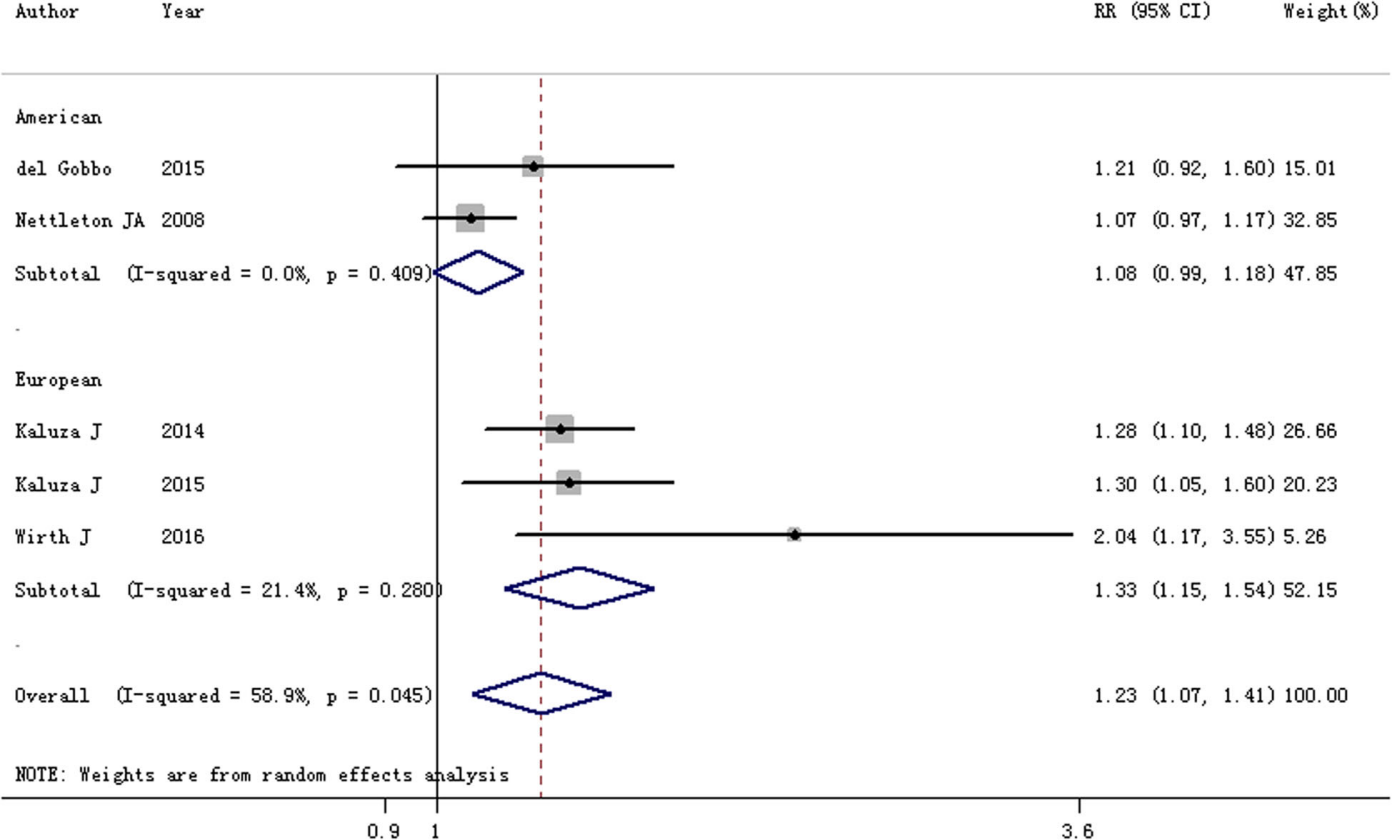
Forest plot of the association between processed meat intake and the risk of heart failure by the subgroup of geographic location

Five studies were applied to assess the association between the processed meat intake and the risk of heart failure. It was reported that two studies presented an increased but non-significant association between the highest processed meat intake and risk of heart failure, while 3 studies demonstrated a positive relationship between processed meat intake and the risk of heart failure. The pooled RR for the highest category of processed meat intake versus lowest intake was 1.23 (95% CI = 1.07–1.41; *I*^2^ = 58.9%, *P* = 0.045; Fig. 2). In the stratified analysis by geographic location, a significant association was only found among the Europeans [RR = 1.33, 95% CI = 1.15–1.54], but not the Americans [RR = 1.08, 95% CI = 0.99–1.18].

In this research, we found high between-study heterogeneity (*I*^2^ = 58.9%, *P* = 0.045) regarding the association between processed meat intake and risk of heart failure. Munafo et al. [27] reported that between-study heterogeneity in meta-analyses is common. Therefore, it is essential to determine if heterogeneity exists between studies. Meta-regression was used for exploring the reasons. We found a significant impact on between-study heterogeneity for covariates of geographic location (*P* = 0.021). The between-study heterogeneity was reduced to zero for the Americans and 21.4% for the Europeans.

The Egger regression asymmetry test (*P* = 0.102) and filled funnel plot (Additional file 1: Figure S1) indicated that publication bias scarcely existed in the meta-analysis. Sensitivity analyses showed that no single study had essential effect on the overall result (Additional file 2: Figure S2).

### Red meat and heart failure risk

**Fig. 3:**
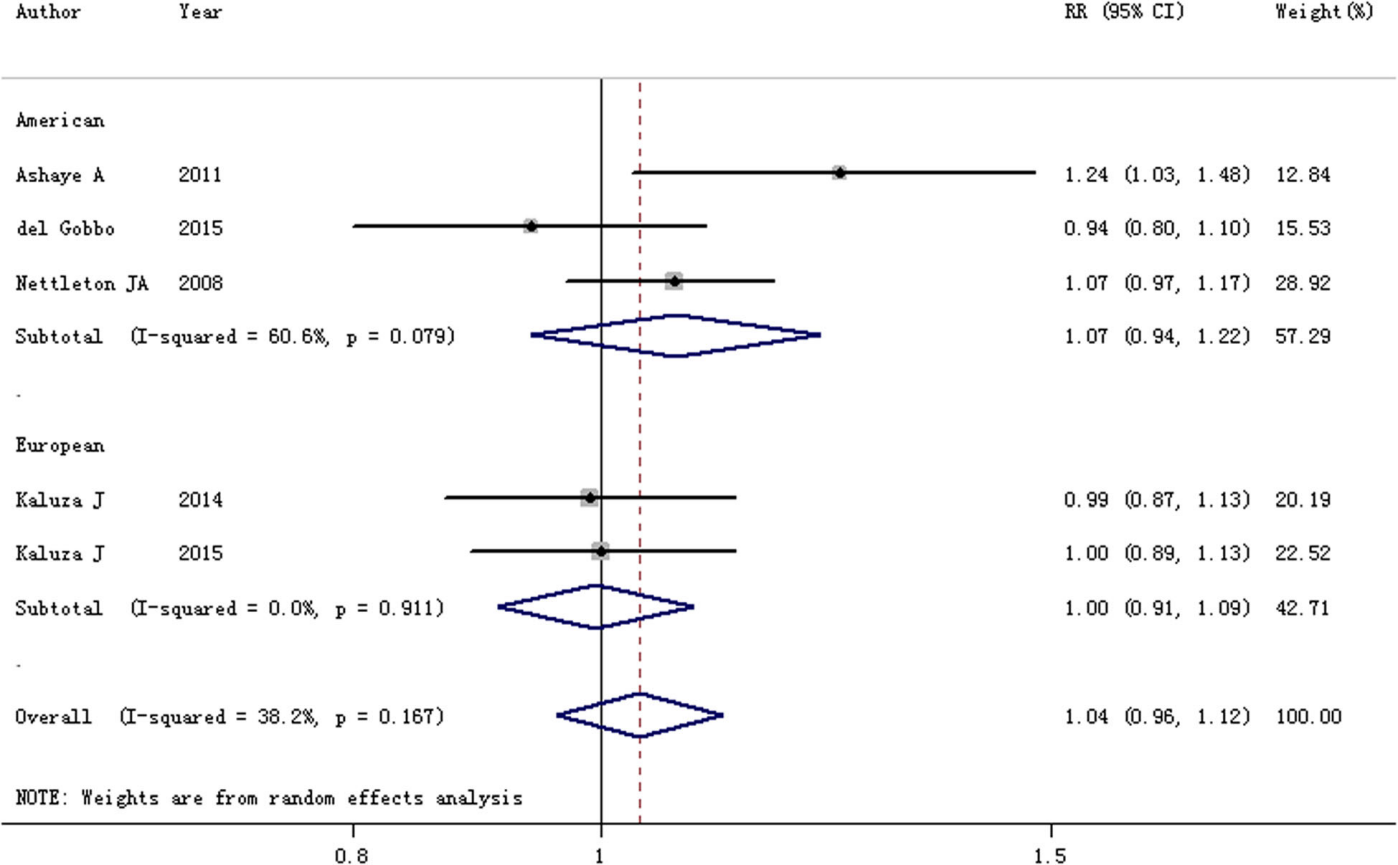
Forest plot of the association between red meat intake and the risk of heart failure by the subgroup of geographic location

Five publications were included to assess the association between red meat intake and the risk of heart failure. Merely one study reported a positive association between red meat intake and risk of heart failure (RR = 1.24, 95% CI = 1.03–1.48), while the remaining 4 studies suggested no significant association between these two. Pooled results pointed out that no statistically significant association exists in the overall studies (RR = 1.04, 95% CI = 0.96–1.12, I^2^ = 38.2%, *P*_heterogeneity_ = 0.167; Fig. 3). The association was not significant in the Americans or the Europeans.

Rare publication bias was detected by using the Egger regression asymmetry test (*P* = 0.221) and filled funnel plot (Additional file 3: Figure S3). No single study affected the aggregate of results when one study at a time was removed (Additional file 4: Figure S4).

## Discussion

This study conducted the most comprehensive analysis of the association between the intake of dietary processed meat and red meat and the risk of heart failure up to date. The overall analyses indicated that the highest category of processed meat intake was associated with increased risk of heart failure. An essential association was found among the Europeans, but not the Americans regarding the processed meat intake. However, the association was not marked between dietary red meat intake and risk of heart failure. Sensitivity analysis demonstrated that no single study is potential to affect the aggregate results when one study was removed at a time.

Recent studies suggested that the highest category of processed meat and red meat raised up the risk of coronary heart disease [28], stroke [29], cardiovascular disease [30], as well as all-cause mortality [31]. However, the present study only found out the highest category of processed meat intake is an increased risk factor for heart failure, but not red meat intake. The potential effect of processed meat on heart failure may be attributed to the higher amounts of sodium and food additives added into the meat during processing procedure. A previous study confirmed that higher dietary sodium intake [14] boosted the risk of heart failure since blood pressure is affected by high sodium intake. Furthermore, processed meat has been used as a marker of N-nitroso compound exposure and reported as a risk factor for heart failure.

In meta-analysis, this study discovered that processed meat intake increase the risk of heart failure among the Europeans, but not the Americans, which might be caused by the fact that the Europeans were inclined to the processed meat with more sodium [14], red meat, and other high-fat, high-calorie, high-cholesterol snacks.

Overall, several strengths characterized this meta-analysis. Firstly, the meta-analysis included the large number of cases and participants than a single study, which may yield more comprehensive results. Secondly, all of the included studies proposed prospective experimental design, which may not cause retrospective or selected bias. Thirdly, the association between risk of heart failure risk and processed meat and red meat intake was verified for the first time.

Some potential limitations in this study should be mentioned. First of all, the included studies in our analysis were merely from Europe and America. Therefore, more original studies that are conducted in other countries are warranted to further evaluate the relationship between meat intake and risk of heart failure. Second, evidence of high heterogeneity was detected in the relationship between processed meat intake and risk of heart failure. Although we extracted the RR with adjustment for most confounding factors from original studies, the between-study heterogeneity was not avoided since some of other exposures associated with consumption of processed meat may be a risk factor for heart failure. However, this high heterogeneity was resolved by meta-regression.

## Conclusion

The findings from this meta-analysis indicated that processed meat intake, other than red meat intake, correlated with an increased risk of heart failure.

## Additional files

Additional file 1: **Figure S1.** Filled funnel plot between processed meat intake and the risk of heart failure. (TIF 25 kb)

Additional file 2: **Figure S2.** Sensitivity analysis between processed meat intake and the risk of heart failure. (TIF 35 kb)

Additional file 3: **Figure S3.** Filled funnel plot between red meat intake and the risk of heart failure. (TIF 25 kb)

Additional file 4: **Figure S4.** Sensitivity analysis between red meat intake and the risk of heart failure. (TIF 36 kb)

## Abbreviations

CI: Confidence interval
NOS: Newcastle-Ottawa-Scale
RR: Relative Risk
