# Supplementary figures and images for: Association between intake of red and processed meat and the risk of heart failure: a meta-analysis
Source: BMC Public Health. 2019 Mar 29;19:354. doi: 10.1186/s12889-019-6653-0 (PMC6440157; doi:10.1186/s12889-019-6653-0)

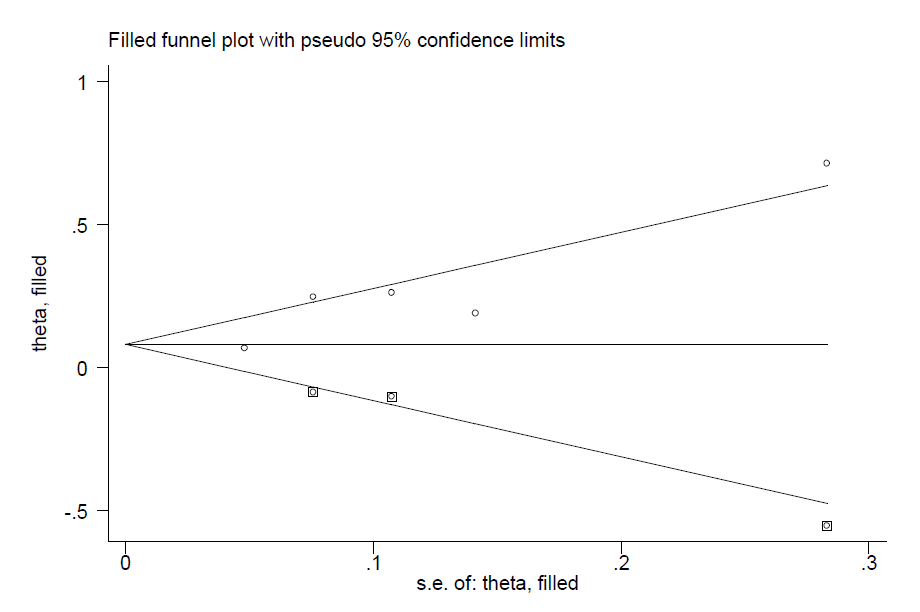

Supplement: Supplementary file 1 — Figure S1. Filled funnel plot between processed meat intake and the risk of heart failure. (TIF 25 kb) [file 12889_2019_6653_MOESM1_ESM.tif]

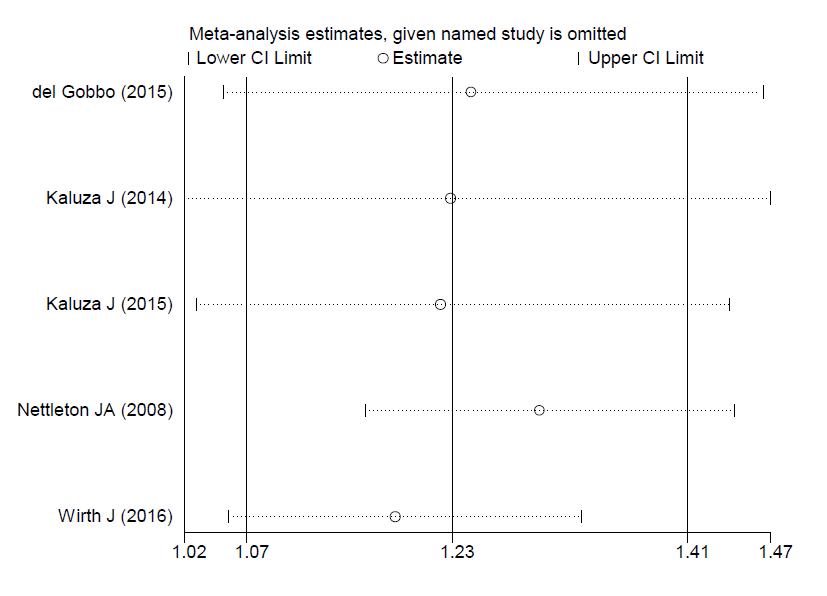

Supplement: Supplementary file 2 — Figure S2. Sensitivity analysis between processed meat intake and the risk of heart failure. (TIF 35 kb) [file 12889_2019_6653_MOESM2_ESM.tif]

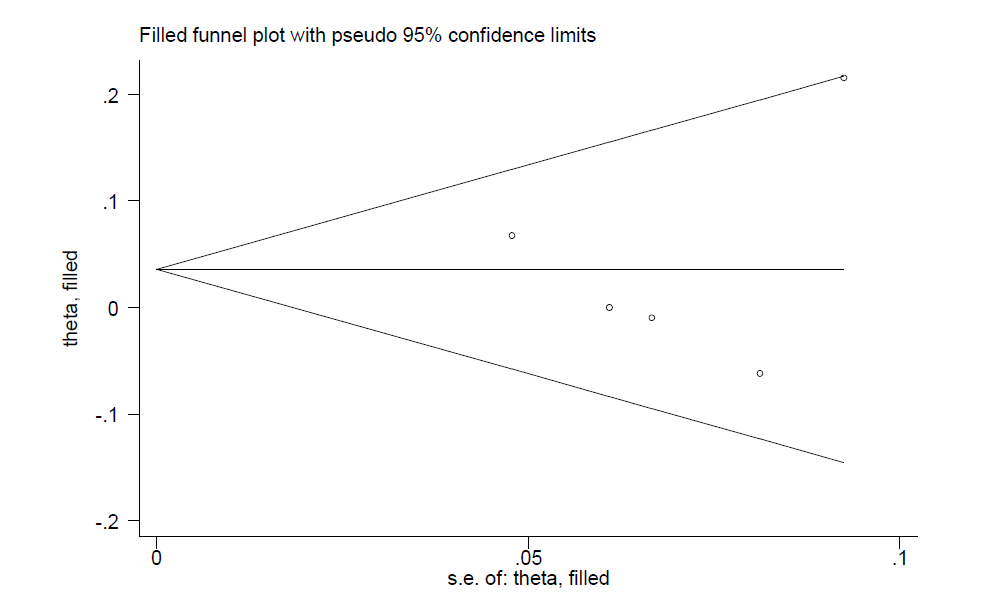

Supplement: Supplementary file 3 — Figure S3. Filled funnel plot between red meat intake and the risk of heart failure. (TIF 25 kb) [file 12889_2019_6653_MOESM3_ESM.tif]

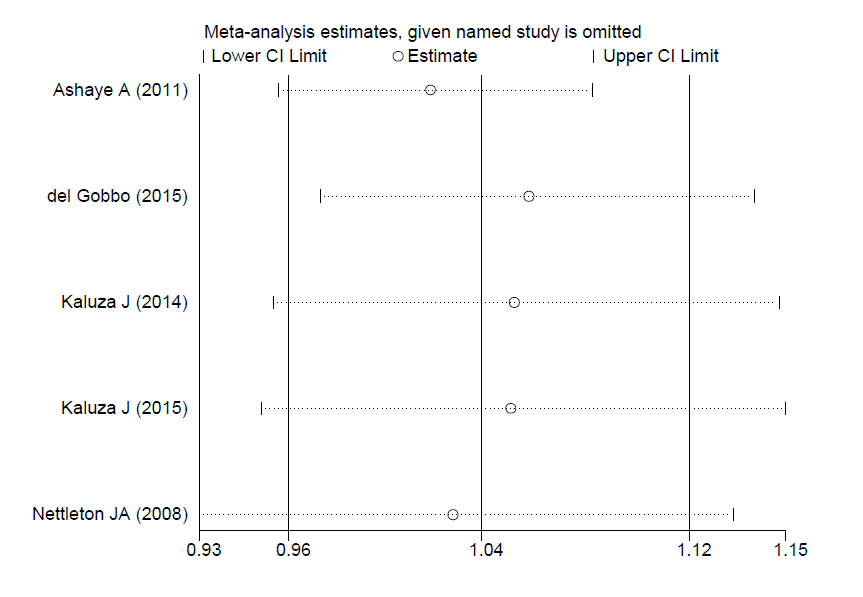

Supplement: Supplementary file 4 — Figure S4. Sensitivity analysis between red meat intake and the risk of heart failure. (TIF 36 kb) [file 12889_2019_6653_MOESM4_ESM.tif]
